# Supplementary material for: Patterns of Intron Gain and Loss in Fungi
Source: PLoS Biol. 2004 Nov 30;2(12):e422. doi: 10.1371/journal.pbio.0020422 (PMC532390; doi:10.1371/journal.pbio.0020422)
Supplement: Table S1 — Also available at http://genes.mit.edu/NielsenEtAl/. (4.3 MB ZIP). [file pbio.0020422.st001.zip › NielsenEtAl/html/340.html]

AN5899.1.NCU07679.1.MG07098.1.FG05105.1


```
 CLUSTAL W (1.82) Multiple Sequence Alignments - Introns Inserted


Sequence 1: NCU07679.1	1179 aa
Sequence 2: MG07098.1	1179 aa
Sequence 3: FG05105.1	1180 aa
Sequence 4: AN5899.1	1179 aa
Alignment Length: 1180 aa
Number Identitical Residues: 794 aa
Alignment Score (without introns) 33420


MG07098.1 	MRVTEIIID0GFKSYAVRTVISGW2DESFNSITGLNGSGKSNILDAICFVLGITNMSTVR
NCU07679.1	MRVTEVIID0GFKSYAVRTVISGW2DESFNSITGLNGSGKSNILDAICFVLGITNMSTVR
FG05105.1 	MRVIEVIID0GFKSYAVRTVISGW2DESFNSITGLNGSGKSNILDAICFVLGITSMATVR
AN5899.1  	MRITEIIID0GFKSYAVRTVISGW2DESFNSITGLNGSGKSNILDAICFVLGITNMSTVR
          	**: *:*** ************** *****************************.*:***

MG07098.1 	AQNQQ0DLIYKRGQAGVTKASVTIVFDNRDKKKSPIGFEEYATISVTRQVVLGGTSKYLI
NCU07679.1	AQNLQ~DLIYKRGQAGVTKASVTIVFDNRDKKKSPIGFEEYATISVTRQIVLGGTSKYLI
FG05105.1 	AQNIQ0DLIYKRGQAGVTKASVTIVFDNRDTKKSPIGFEEYATISVTRQIVLGGTSKYLI
AN5899.1  	AQNLQ~DLIYKRGQAGVTKASVTIVFDNRDTAKSPIGFEEYATISVTRQIVLGGTSKYLI
          	*** * ************************. *****************:**********

MG07098.1 	NGHRAQQQTVQNLFQSVQLNINNPNFLIMQGRITKVLNMKAVEILAMIEEAAGTRMFEDR
NCU07679.1	NGHRAQQQTVQNLFQSVQLNINNPNFLIMQGRITKVLNMKAVEILAMIEEAAGTRMFEDR
FG05105.1 	NGHRAQQQTVQNLFQSVQLNINSPNFLIMQGRITKVLNMKAVEILAMIEEAAGTRMFEDR
AN5899.1  	NGHRAQQQTVQNLFQSVQLNINNPNFLIMQGRITKVLNMKAVEILSMIEEAAGTRMFEDR
          	**********************.**********************:**************

MG07098.1 	RDKALKTMAKKEMKLQEIKALLSEEIEPKLEKLRTEKRAFLDFQQTQNDLERLTRVVVAH
NCU07679.1	RDKALKTMAKKEMKLQEIKELLRDEIEPKLEKLRTEKRAFLDFQQTQNDLERLTRLVVAH
FG05105.1 	RDKAIKTMAKKETKLVELKELLKDEIEPKLEKLRTEKRAFLDFQQTQNDLERLTRVVVAY
AN5899.1  	REKAAKTMAKKDLKLQEIEGLLKEEIEPKLEKLRAEKRAFLDFQQTQNDLERLTRLVVAH
          	*:** ******: ** *:: ** :**********:********************:***:

MG07098.1 	DYVRCLEKLQQSAADLEAKKQRRKDLEDSALKLKSEIAHLEEDVMRVKEQRDKELRKGAK
NCU07679.1	DYVRCQEKLQQSASDLEAKKQRQKELEESAIRLKNEISHLEEDLQRVKAQRDKELRKGGK
FG05105.1 	DYLRYQDSLSQSAADLEGKKQRQRDLEDSAARLKSEINHLEEDAKKVRAHRDKELRKGGK
AN5899.1  	DYLRSGDRLRASGEECEKKKRKIQALEDNTAKLKSEIAHMEEDVKRVKAVRDKELRKGGK
          	**:*  : *  *. : * **:: : **:.: :**.** *:***  :*:  ********.*

MG07098.1 	AHALEEEAKRHSNELVRLATVVDLKKSSMAEEEERRAAGEKSVTELEAALEEKTKAYEKV
NCU07679.1	AQALEEAVKKHANELVRLATVVDLKKSSMKEEQERRKAGEKNVADLEAALKEKTKTYEKI
FG05105.1 	ASELEEAAKKHSNELVRLATILDLKKSSLAEEEEKKLAVERTVSELEATLEDKTAAFENA
AN5899.1  	FQALEDEVKAHSHELVRLTTVFDLKKASIAEEKEKSKELQKSVKDLETSLKEKKKIYDKL
          	   **: .* *::*****:*:.****:*: **:*:    ::.* :**::*::*.  ::: 

MG07098.1 	KAKYDLASEQLEKQRRDAEAKEELLQTLQTGVASKEGQENGYQGQLQDARNRATTAMTEQ
NCU07679.1	KAKYDAAKETLEKQRQEADTKEELLQTLQTGVASKEGQENGYQGQLQDARNRATAAATEQ
FG05105.1 	KARYDAAKEDLEQQNKDAESKEELLQTLQTGVASKDGQENGYQGQLQDAKNRATTAATEQ
AN5899.1  	QAEYDAAKAELDAQTAEVEQKEELLQTLQTGVASKEGQESGYQGQLQDARNRASNAATEQ
          	:*.** *.  *: *  :.: ***************:***.*********:***: * ***

MG07098.1 	EQAKIKITHLEKRIKEEEPRAKKAKTQNAGLLRDLDGLKSQAQRLEKELGKLGFSPGAEE
NCU07679.1	EQAKIKIAHLEKRIKGEEPRARKAKEQNAGLLKDLDGLKAQAQRLEKELGRLGFQPGTEE
FG05105.1 	EQAKIKIAHLEKRIREEEPRAEKAKVQNADLLRDLDGLKVQAQKLEKELSRLGFEPGQEE
AN5899.1  	EQAKLKIAHLEKRIKEEEPRAKKAKEQNSGLLKELEGLKSQAKKLEYELSKLGFEPGREE
          	****:**:******: *****.*** **:.**::*:*** **::** **.:***.** **

MG07098.1 	DMYKKESSLQQSIRGLRQESDALKRKVANIDFNYSDPVANFDRSKVKGLVAQLFTLDKNQ
NCU07679.1	EMYKQESSLQQTIRNLRQEADALKRKVANIDFNYADPVPNFDRSKVKGLVAQLFSLPKEY
FG05105.1 	QMYKQESELQQTVRGLRQESDKLKRQVANTEFNYADPVPNFDRSKVKGLIAQLFTLDESY
AN5899.1  	QLYQEQSGLQKEIRELRQRADGLQRKVANIEFNYSDPYPNFDRSKVKGLVAQLFTVDKEK
          	::*:::* **: :* ***.:* *:*:*** :***:** .**********:****:: :. 

MG07098.1 	TRAGTALEICAGGRLYNVVVDNSVTGTQLLQNGKLRKRVTIIPLNKIAAFKASAATIATA
NCU07679.1	MEAGTALEICAGGRLYNVVVDTEVTGTQLLQGGRLRKRVTIIPLNKISAFKASAQTIANA
FG05105.1 	TQAATALEICAGGRLYNVVVDSEVTGTQLLQRGKLRKRVTIIPLNKIAAFKASAQTIATA
AN5899.1  	LQAATALEICAGGRLYNVVVDSSDTGTQLLQKGKLRKRVTIIPLNKISAFRASVEKIGAA
          	 .*.*****************.. ******* *:*************:**:**. .*. *

MG07098.1 	QKIAPGKVDLALSLVGYDEEVSAAMEYVFGNTLICADAETAKRVTFDPNVRMRSITLEGD
NCU07679.1	QRIAPGKVHLALSLVGYDDEVSAAMDYVFGNTLICADADTAKRVTFDPNVRMRSITVEGD
FG05105.1 	QNIAPNKVDLALSLVGYDDEVSAAMEFVFGNTLICADADTAKRVTFDPKVRMRSITLEGD
AN5899.1  	QNLAPGKVDLALSLIGYDEEITAAMNYVFGNTLIANDAETAKRVTFDPSVRIKSVTLDGD
          	*.:**.**.*****:***:*::***::*******. **:*********.**::*:*::**

MG07098.1 	AYDPSGTLSGGSAPNSSGVLVTLQKLNEINKNIKEAEAALGELQAMISREKSKLDQARSL
NCU07679.1	SYDPSGTLSGGSAPNSSGVLVTLQKLNEINRQLKEAETALNELRGQIYREKTKLDQAKRI
FG05105.1 	AYDPSGTLSGGSSPNSSGVLVLLQKLNGLTRQLSEAESTLRELQARISNEKAKLDHARKI
AN5899.1  	VYDPSGTLSGGSAPNSSGVLVTLQKLNEITREIRSKERVLAALEETMKKEKKKLDAVRSI
          	 ***********:******** ***** :.::: . * .*  *.  : .** *** .: :

MG07098.1 	KQELDLKTHEIKLTEEQIGGNSSSS~IIQEVENMKEQIVQLKEGVSDAKKRQAEANADVK
NCU07679.1	KQELDLKAHEIKLAEEQIGGNSSSS~IIQEIANMKETIVQLKQSIMEAKQRQAEATAEAK
FG05105.1 	KQGLDLKTHEIKLAEEQISGNSSSS~IIQEVENMKSTIKELKESIAEAKTRQAKASADVK
AN5899.1  	KQELDLKNHEIKLTEEQIGGNSSSS0IIQAVEEMKANIEQLKQNISDAKLRQSEASKDIK
          	** **** *****:****.****** *** : :**  * :**:.: :** **::*. : *

MG07098.1 	RIEKDMNDFDNNKDAKLVELQSSLKQLQSLLSANAASVKTLQKELQGAQLDSEQAGGDLA
NCU07679.1	RIEKDMKDFDNNKDAKLVELQASVDKLRASLDKMSVTNKALQKELQEAQLDSEQVAADLA
FG05105.1 	TIEKDMKDFDNNKDGKLVELQKALDKLRAGLGKNAAAVKTLQKELQGAQLDSEQAGFDLS
AN5899.1  	RIEKDMREFNNNKDSKLEELQTTLNALKKSLSKNLTSVKNLHKELQASRLESEQVGSDLS
          	 *****.:*:****.** *** ::. *:  *.   .: * *:**** ::*:***.. **:

MG07098.1 	AAREQLQDIDLAIKAQKEEIADLNNQQAALK~ETHDAAQAKLDEERAKLSIYDDELHALE
NCU07679.1	AAREQVQDIDVALKAQQEEIDDIVKQGTVLQ~ETHDAVLAQLEDERTKLHVYDDELRALE
FG05105.1 	AAREQLQEVEVAIKAQQKDIEDLNKQKAELT~ETHDTVQAELDDERAKLHQFDDELRALD
AN5899.1  	AAEEQYAESESTLKAQLEEVDSLKREQARIK0DAHDIAQAHLDDERAKLTGFDDELRDLE
          	**.**  : : ::*** ::: .: .: : :  ::** . *.*::**:**  :****: *:

MG07098.1 	EATRSKNSRIAEEGLEMQQLGHQVDKFHKEQQAAGQMAANMEKEHDWIVDEKDRFGRSGT
NCU07679.1	DATRSKNARIAEEGLEMQKLGHQIEKFHKEQQQAAQTASHMEREHDWIAETKDQFGRPGT
FG05105.1 	DATRSKNARIAEESLEMQKLVHLLEKFNKEQQGAAEKVARLEREFDWIADEKDNFGRSGT
AN5899.1  	QTMQSKNSQITEEGLEMQKLGHQLEKLQKEQNAAEQAVAHLEAEHEWIADEKDNFGRPNT
          	:: :***::*:**.****:* * ::*::***: * : .:.:* *.:**.: **.***..*

MG07098.1 	PYDFKGQNIGEAKATLKNLTERFQGMKKKINPKVMNMIDSVEKKELSLKHMMKTVIRDKR
NCU07679.1	LYDFKGQNIAECKSTLRNLTERSQGLRKKINPKVMNMIDSVEKKEVSLKHMMRTVIRDKR
FG05105.1 	PYDFKDHNIGECKATLHNLTERFQGMKKKINPKVMNMIDSVEKKEVSLKHMIKTVIRDKR
AN5899.1  	VYDFKNQNIAECKATLRNVTERFQGMKKKINPKVMNMIDSVEKKEAALKNMMRTVIRDKS
          	 ****.:**.*.*:**:*:*** **::****************** :**:*::****** 

MG07098.1 	KIEETIVSLDDYKKKQLQETWEKVNADFGQIFAELLPG-SFAKLDPPEGKTISDGLEVKV
NCU07679.1	KIEETILSLDDYKKKALQETWEKVNADFGQIFAELLPG-SFAKLDPPEGKTISEGLEVKV
FG05105.1 	KIEETIVSLDDYKKKALHETWEKVNGDFGNIFSELLPGGSFAKLDPPEGKTISEGLEVKV
AN5899.1  	KIEETIINLNEYKKEALHKTWTKVNADFGQIFSELLPG-SFAKLDPPEGKDITDGLEVKV
          	******:.*::***: *::** ***.***:**:***** *********** *::******

MG07098.1 	SLGKVWKQSLTELSGGQR2SLIALSLIMALLQFKPAPMYILDEVDAALDLSHTQNIGRLI
NCU07679.1	QLGKVWKQSLTELSGGQR2SLIALSLIMALLQFKPAPMYILDEVDAALDLSHTQNIGRLI
FG05105.1 	CLGKVWKQSLTELSGGQR2SLVALSLIMALLQFKPAPMYILDEVDAALDLSHTQNIGRLI
AN5899.1  	SLGKVWKQSLTELSGGQR~SLIALSLIMALLQFKPAPMYILDEVDAALDLSHTQNIGRLI
          	 ***************** **:**************************************

MG07098.1 	KTRFKGSQFIVVSLKDGMFQNANRIFRTRFSEGTSMVQALTPADMR
NCU07679.1	KTRFKGSQFIVVSLKDGMFQNANRIFRTRFSEGTSMVQALTPADLK
FG05105.1 	KTRFKGSQFIVVSLKDGMFQNANRIFRTRFSEGTSMVQALTPADMK
AN5899.1  	KTRFKGSQFIVVSLKDGMFQNANRIFRTRFSEGTSVVQALTPADLR
          	***********************************:********::
```
